# Every good biologist deserves favour

**DOI:** 10.1098/rsob.220368

**Published:** 2023-01-11

**Authors:** Jon Pines

**Affiliations:** Division of Cancer Biology, Institute of Cancer Research, London SW7 3RP, UK

Despite first appearances, this will not be an editorial about melody or mnemonics, but about two new initiatives for 2023. These two initiatives go to the heart of science: integrity and constructive dialogue. Science absolutely depends on integrity—we need to trust that published data are genuine or our whole enterprise fails. Regrettably, this axiom is not always recognised; like many journals, *Open Biology* has had to put in place measures to check the veracity of submitted data and guard against fraudulent papers https://royalsocietypublishing.org/doi/10.1098/rsob.200165. This year, we will go further to ensure research integrity by collaborating with other publishers as part of the STM Integrity Hub (https://www.stm-assoc.org/stm-integrity-hub/) to share intelligence and best practices. We will also disseminate the importance of integrity and ethical behaviour to potential authors through Research Integrity seminars and training forums, particularly to highlight the insidious practice of papermills.

Progress in science is equally dependent on constructive dialogue: discussions with knowledgeable colleagues help us to clarify our ideas, put them in context and interpret our results from a different perspective. This is the value of peer review, in addition to upholding standards of experimental design and quality of data, but done properly it takes considerable time and effort on the part of reviewers. We recognise this, and to show our appreciation in concrete terms we will be rewarding our reviewers with tokens that can be put towards open access charges for future publications. We hope that this will encourage more people to agree to contribute their valuable time and expertise as reviewers for *Open Biology*.

We are also very grateful for the time and effort contributed by our editorial board in handling papers and upholding our journal policies and ethical standards. Special thanks go to our enthusiastic Preprint Team led by our Preprint Editor Michael Ginger, whose goals are to broaden the scope of articles we publish and the visibility of *Open Biology*. The team have helped to raise the profile of preprint servers, which are an increasingly important conduit for disseminating and enabling equitable access to scientific research.

We hope that our initiatives will help to strengthen the reputation of *Open Biology* as a home for rigorous science, where peer review serves its real purpose to improve the science we publish. But we also want *Open Biology* to be a place for exciting ideas, so I encourage you to take advantage of our ‘Opening Up’ section in your paper to discuss the wider implications of your discoveries, and our ‘Open Questions’ format to bring unresolved or contentious issues to a wider audience.

Thus, *Open Biology* will reward good behaviour on the part of both authors and reviewers, and we all will benefit.

